# Mathematical Method of Rational Placement of Gas Fire Sensors †

**DOI:** 10.3390/s23208349

**Published:** 2023-10-10

**Authors:** Andrey Petrov

**Affiliations:** Department of Computer-Aided Engineering and Design, National University of Science and Technology MISIS (NUST MISIS), 4, Leninsky Prospect, Moscow 119049, Russia; petrov.ae@misis.ru

**Keywords:** gas sensors, mathematical model, rational location, automated system, early fire detection, gas control systems

## Abstract

Ensuring fire safety is an important condition for the successful operation of industrial enterprises. It is necessary to detect the threat of fire before the ignition. An indicator of danger is the appearance of gas, which occurs as a result of unauthorized heating of the equipment. Gas sensors make it possible to detect the appearance of gases characteristic for the decomposition of materials at the stage of smouldering and pyrolysis, which cause a change in the composition of the atmosphere. This paper presents a mathematical method for the rational placement of gas analyzers in the premises of potentially hazardous industries. The rational placement of gas sensors ensures safety, taking into account economic feasibility. For the first time, an analysis of the accumulation of gas flow from the source to the ceiling, and then to the location of the sensors, was used to select the location of gas sensors. Depending on the permissible values of the gas volume, the placement and number of gas sensors are selected. The calculation of the conditions for the rational placement of gas sensors is carried out according to the most common indicators, such as carbon monoxide and hydrogen at different sizes and heights of premises.

## 1. Introduction

Timely detection of fire hazards is a critical condition for ensuring safety in agriculture, industry and the environment. In systems for early detection of fire hazards in industry, highly sensitive gas analyzers are used, in which sensors ensure the fixation of trace concentrations of hazardous gases. They are used to detect the possibility of ignition by gas emission at the stage of smouldering (decomposition) and pyrolysis (overheating), i.e., before the appearance of an open flame.

Most of the combustible substances are organic compounds, the smouldering or combustion of which emits carbon dioxide CO_2_ and carbon monoxide CO. The sensor is a sensitive element of the gas detector, which registers an increase in the concentration of these gases and gives a signal that the specified limit of the controlled parameter has exceeded. When smouldering, the concentration of CO increases from 20 to 100 mg/m^3^, but when a flame appears, it decreases. Also, during the smouldering of organic materials, hydrogen, H_2_, is released, the concentration of which, up to 10 mg/m^3^, can be detected in the presence of semiconductor sensors. When a small amount of material smoulders, the concentration of hazardous gases is small and they are distributed in the volume of the room due to diffusion. For gas detectors reacting at this stage of the fire, the requirement for the sensitivity threshold of the sensors is from 0.0001% for CO and 0.00001% for H_2_ [1,2].

The installation of gas control systems in the protected room makes it possible to detect the appearance of gases characteristic for the decomposition of organic materials, including insulation of cables, packaging, wood, fabrics and much more [3]. Thermal degradation in different materials begins at different temperatures, but it is ahead of the appearance of smoke. The heating process is slow. At the same time, the physical principles underlying the operation of the fire sensors used (temperature increase, light scattering by smoke particles, ion current due to radioactive ionization of smoke) reliably register a fire already at the combustion stage when an open flame appears, which is accompanied by a strong change in the physical properties of the surrounding air [4]. A fire at the open flame stage cannot be eliminated by technical measures by changing the operating mode, for example, turning off the equipment.

A reliable way to prevent a fire at an early stage is to control the chemical composition of the air, which changes dramatically due to thermal decomposition, pyrolysis, overheating and the smouldering of combustible materials [5]. It is at this stage of the development of the fire that the necessary measures can be taken to extinguish it, and the in case of the overheating of electrical equipment and cables, they can be automatically turned off in time by a gas sensor signal.

Gas sensors are used all over the world in various fields as fire sensors to improve the reliability of fire safety systems [6,7,8,9,10,11,12]. However, the developed norms and guidelines do not consider methods for the rational arrangement of sensors. A set of rules for the design of fire protection systems was developed by the Ministry of Emergency Situations of Russia for timely and reliable fire detection, providing information to the personnel on duty [6]. The placement of gas analyzers for plant protection systems and life safety is presented in [7]; guidelines for the use of stationary or portable gas detectors are proposed in [8]. The use of gas sensors in industry, as well as in other areas, is considered in [9,10]. The application of gas detectors for real-time detection of chemicals in the workplace is discussed in [11]. The use of gas sensors for stationary and portable gas detection in industrial processes associated with the use and production of hazardous substances, especially toxic and combustible gases, is discussed in [12].

Guidance on the placement of individual sensors of combustible gases in rooms is given in [13]. The main recommendations for the selection of fire and gas detectors are presented in [14]. The placement of sensors in industrial premises is not regulated in these rules and recommendations. In [15], modeling is used to optimize the location of sensors for early detection of a gas cloud. In operation [16], the system with the microcontroller should detect a gas leak, issue a warning via SMS, and turn on the fire extinguishing system. A formulation of nonlinear programming with a mixed integer was proposed for the optimal placement of flame detectors, taking into account the non-uniform probabilities of detection failure [17]. An approach to a fire detection system in valuable forests is proposed, which can detect fires from the earliest stage using a mathematical model of the detector geometry [18].

Convolutional neural networks are used to detect fire based on aerial photography [19]. The results of experimental investigations of the main characteristics of a typical indoor seat of fire at the stages of initiating the ignition of combustible materials and in the process of their burning are presented in [20]. Wood material, wood fiberboard, linoleum, and plastic panels have been used as combustible materials.

A holistic approach to fire safety design, which describes fire models, is discussed in [21]. An attempt to create an integrated automated system for the early detection of fires and environmental monitoring was made in [22]. Gas sensors and methods of their placement are necessary to ensure the fire safety of underground structures [23].

The author has developed network models for analyzing the flows of hazardous products to ensure the safety of oil refineries with practical application at the Moscow Refinery and the Ryazan Refinery [24]. A network of branches is a tensor; different connections of branches and structures are considered as projections of the tensor into different coordinate systems. Coordinates are paths, lines passing through branches. Closed and open paths form orthogonal subspaces in the tensor network, the dimension of which changes as the connections change. In a dual network, connections correspond to breaks, and vice versa. The sum of the dimensions of the subspaces of closed and open paths in the two networks remains constant. The duality invariant connects the transformations of the structure [25].

The duality of the networks made it possible to represent mass flows in the distillation column as a contour network with a basis of closed paths, and heat flows as a nodal network with a basis for open paths. This made it possible to propose the placement of pressure sensors in the branches, which determine the basic closed paths in the installation. Temperature sensors will place in the elements that determine the basic open paths. The analysis shows where the maximum permissible values are exceeded when the branches are broken, which simulates accidents in the installation.

In all cases, with the actual size of the production, it is necessary to place the sensors, taking into account the requirements and conditions of ensuring safety.

The article presents a method for rational placement of sensors in production conditions for early warning of a possible fire. The method makes it possible to create sensor placement schemes, depending on changes in the size, volume and shape of protected premises, types of production, creation of new sensors, and changes in the composition of hazardous gases in different technological processes. The method allows you to adjust sensor technologies by changing the layout, depending on the increase in the sensitivity of the sensors, the shape and size of the room, and the requirements for the maximum permissible volumes of hazardous gases at the stages of smoldering, before the occurrence of a fire.

It is assumed that in case of malfunctions and heating, dangerous light gases appear; for example, CO and H_2_ rise to the ceiling of the room where the sensors are located. The physical foundations of the first stage of the process of gas propagation from the heating source to the ceiling of the room were considered in [26,27]. The volume of gas accumulated during the ascent spreads along the ceiling reaches the sensors, which give a signal about the danger and the need to take measures to prevent fire. According to the coordinates of the sensors that gave a signal about the appearance of hazardous gases, it is possible to localize the heating source and automate the warning of a possible fire.

## 2. Location Methods for Gas Fire Sensors

### 2.1. Problem Statement

The volume of the production room is filled with air. The boundaries of the room are the floor, the ceiling and the walls. In the room there is equipment that performs a technological process with a potential fire hazard. A harbinger of fire is the appearance of gas, which occurs as a result of unauthorized heating of the equipment or premises.

It is necessary to place gas fire sensors (GFS), which will record the appearance of indicator gases such as H_2_ and CO. The sensor signal will allow you to determine the possible localization of combustion products in the room to eliminate heat sources and prevent fire, or take measures to extinguish the fire. The H_2_ and CO gases that appear as a result of heating are lighter than air, and therefore, will rise upwards. In addition, the density of the heated gas is less than the density of the air, so it is affected by a lifting force equal to the weight of the volume of air displaced by the gas.

It is assumed that the most likely sources of gas are in the area of the floor where the equipment is located, and there is a risk of fire or explosion. The controlled gases propagate upwards and to the sides, so the gas sensors must be positioned at the top in such a way as to ensure that low concentrations of H_2_ and CO are recorded before the concentration of the gas emitted reaches a dangerous value.

There are various configuration options for gas sources (emissions):

- At the point;
- At several points;
- In the direction of the line (crack);
- On a surface area (surface rupture).

The release of gas through a line or surface can be thought of as a generalization of the release through a point. It can be assumed that the air in the room is stationary (or its movement can be neglected), and the air is “transparent” to the spread of gas.

The placement of sensors should ensure contact with gas molecules when the accumulated volume does not exceed the maximum permissible values set by safety standards.

### 2.2. Methods of Placement of Gas Sensors

There are various methods to solve this problem. For example, you can consider the physics of the process from the standpoint of the location of gas sensors. The power density methods and the location of the lamps solve the problem from the perspective of the location of the gas sensors. It is assumed that the gas analyzer has a “range”. The point method uses analogies with the luminous flux reaching the room areas that need to be illuminated. The disadvantage of these methods is the assumption that the GFS itself reaches the place of possible occurrence of danger.

In reality, the gas analyzer is in place, waiting for the molecules of the substance to enter the inlet in order to then send a signal to the security system. For this reason, this paper uses the method of considering the movement of hazardous gas molecules from the place of danger in the direction of gas sensors.

The challenge is to determine how many gas sensors are needed and how to place them in order to detect and locate possible gas emission sites in a timely manner and monitor the permissible hazardous concentration. This method of placing the GFS solves the problem from the position of gas movement from sources near the floor of the room where the equipment operates, in the direction of the location of gas sensors on the ceiling of the room.

### 2.3. Physical Model of the Gas Movement Process

Gas can appear as a result of heating the equipment located below, so the gas will rise up. The density of the heated gas is less than the density of the air, so it is affected by a lifting force equal to the weight of the volume of air displaced by the gas. Practice shows that, in the source, the concentration (dynamic ratio) can be 300–350 ppm (parts per million), and on the ceiling, at the height of H = 5–6 m, the concentration will be 80–90 ppm. In this case, an approximate concentration gradient is set, which can be considered as a potential difference.

Let the heat source heat the solid material, converting it into a gaseous state. The pressure acts the same in all directions, but under the influence of heating, the gas mainly moves upwards, creating convection currents. There is a stream of gas molecules that occupy a certain volume at the heating site at the floor level. Let us assume that the source has a constant intensity, i.e., the same number of gas molecules appear every second. At constant pressure, the molecules occupy the same volume at any given moment, creating a cloud of gas step by step.

As the gas cloud propagates, it moves upward. Random factors (convection, turbulence, etc.) give the gas cloud a configuration that is difficult to predict. With a high degree of certainty, which is sufficient to solve the problem, we will assume that the gas cloud has the shape of a cone. In the process of movement, the cone occupies all new layers with a vertex at the ejection point. Rising, the gas cloud will touch the ceiling of the room, and then spread along the ceiling in all directions, falling into the GFS location areas.

The gas cloud will spread across the ceiling and reach one or more gas sensors, which will signal the presence of gas in the room. According to the coordinates of the SFS (gas sensors) that gave the signal, it is possible to solve the inverse problem of determining the location of the gas source on the floor.

Thus, the three-dimensional problem is reduced to a two-dimensional problem. The circle of gas on the ceiling that reaches the sensors must have the square that corresponds to the volume of gas less than the maximum permissible value.

The leading edge of the gas cloud on the ceiling will reach one or more gas sensors, which will signal the gas presence in the room. According to the GFS (gas sensors) coordinates that signaled, it is possible to solve the inverse problem in order to determine the location of the gas source on the floor. Thus, a three-dimensional problem is reduced to a two-dimensional problem—the need to rationally place the GFS on the room ceiling so that they are touched by the boundary of the gas propagation circle of a certain diameter.

The analysis shows that at an equal density in the gas cloud, the propagation rate changes in proportion to the cubic root of the time elapsed since the appearance of the source. With this dependence, the rate of gas propagation decreases rapidly with distance from the source.

#### 2.3.1. Movement of Gas from the Source of Danger to the Ceiling of the Room

Heating or a chemical reaction converts a substance that can cause a fire from a liquid or solid state to a gaseous state. The gas occupies a larger volume than the source substance, and this excess, which creates pressure, begins to push the gas molecules into the environment. They penetrate between air molecules, spreading throughout the room. The mass of the gas *m_g_* ejected in the first time interval (per second) is equal to the density of the gas *q* multiplied by the volume of the cone *V* (the radius of the base *r* and the height *h*) that the gas will occupy.

*m_g_* = *V q* = 1/3 *π r*^2^
*h q* (1)

where:

*m_g_*—gas mass, mg;
*V*—cone volume, m^3^;
*r*—cone base radius, m;
*h*—cone height, m;
*q*—gas density, mg/m^3^.

The concentration unit is ppm (parts per million). With a constant source power, the same gas mass *m_g_* will be emitted in each unit of time. The main parameters reflecting the state of the gas are the dynamic pressure *P*, temperature and density *q*. In this case, the task is to determine the release of gas and the appearance of hazardous substance molecules. Thus, the physical effect of a fire hazardous process is the dynamic pressure created as a result of the emergence and propagation of gas molecules. In addition, the temperature decreases from the source to the GFS location.

The gas is formed as a result of pyrolysis (thermal decomposition). Heating can lead to fire, explosion, etc. The heated exhaust gas (H_2_, CO) tends to rise. The pressure on the gas acts in all directions. In addition, the gas molecules are affected by heating and buoyant force directed upwards, so the gas cloud takes on an upward shape. With a good approximation, we can assume that this is the shape of a cone. The top of the cone at each moment of time rests on a source of dangerous gas, where pressure is created.

As time passes, the gas occupies new layers of the truncated cone; let the source have a constant intensity, i.e., emits the same number of mg molecules in each time interval, then, the layers of the cone have the same volumes for each time interval. The density of the gas during propagation remains constant *q* = const. This is a stronger condition than the assumption that the density gradually decreases as it moves towards the ceiling. Let us consider a change in the gas velocity under the condition of a constant flow of molecules emanating from the source and a constant gas density in a gas cloud.

**Figure 1:**
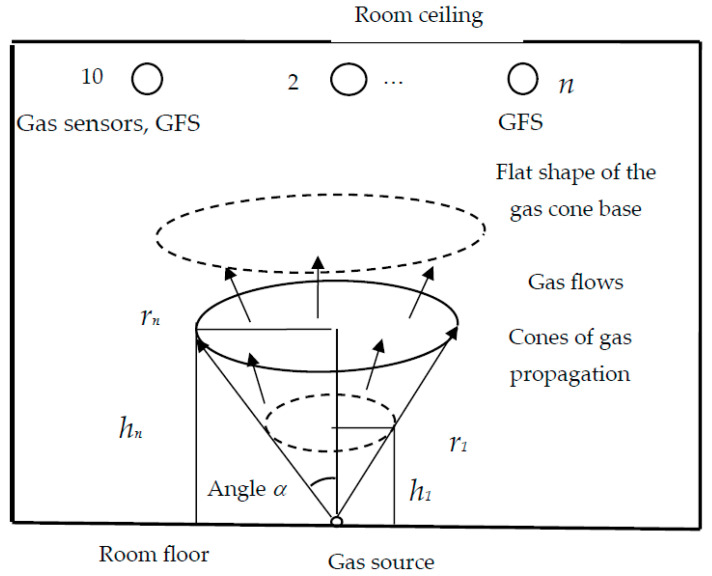
Gas distribution from a source located on the room floor. The upper base of the gas cloud cone is flat.

Suppose that in the lower part of the room, near the floor, the pyrolysis process has begun due to heating, or for other reasons. The source has a local character, which can be represented as a point (see Figure 1). Under the influence of pressure, gas molecules spread evenly, creating a cone-shaped cloud, penetrating between air molecules, displacing them, as well as each other. It is assumed that the intensity of the source is constant. For this reason, the number of molecules in all layers of the gas cone per unit time must be the same.

The cone volume occupied by the gas at the first moment of time will be:

*V*_1_ = 1/3 *h*_1_
*π*
*r*_1_^2^ = 1/3 *h*_1_ *S*,

where *h*_1_—cone height, *r*_1_—cone base radius, *S*—cone base area.

As you can see in Figure 1, the cone radius is equal to the height multiplied by the tangent of the angle *α* (equal to half the angle of the apex of the cone). Then, the volume of the cone *V* can be expressed in terms of the cone height *h* and the angle *α* [27].

*V* = 1/3 *π*
*h*^3^
*tg*^2^
*α*.

The cone height is the distance traveled by the leading front of the gas per unit time, which is numerically equal to the velocity of gas propagation in the first period. Strictly speaking, for this reason, if the cone top is located in the source at the bottom, then the base at the top should have a convex spherical shape.

During the second period, per unit of time, the gas will spread further, occupying the next layer of the cone. The volume of this layer is equal to the volume created in the first unit of time. From here you can find out the estimated speed of gas propagation. Each layer of the gas volume increment has the shape of a truncated cone.

Let the angle of the cone *α* = 45°. During the second period, the gas will occupy the volume *V*_2_. Since the power of the source is constant, then the volume increment, the layer *dV*_2_, which the gas occupies in the second unit of time is equal to the volume that the gas occupied in the first unit of time *V*_1_. The volume increment layer *dV*_2_ has the shape of a truncated cone. Its volume is equal to the difference between the volume *V*_2_ *=* 1/3 *π r*_2_^3^ *=* 1/3 *π h*_2_^3^ and the volume of the cone *V*_1_ *=* 1/3*π r*_1_^3^ *=* 1/3 *π h*_1_^3^. Then, one can write that

*dV*_2_ = *V*_2_ − *V*_1_ = 1/3 *π r*_2_^3^ − 1/3 *π r*_1_^3^ = *V*_1_, (2)

where *dV*_2_ is the increase in the volume of gas propagation in the second unit of time; hence we obtain *V*_2_ *=* 2 *V*_1_ *=* 2/3 *π r*_1_^3^.

Hence, it follows that the radius of the truncated cone *r*_2_ is equal to

(3) $$r_{2}=r_{1}∛2\;or\;as\;r=h,\;then\;h_{2}=h_{1}∛2$$

Similarly, we obtain that the radius of the third cone *r*_3_ is equal to

(4) $$r_{3}=r_{1}∛3\;or,\;as\;r=h,\;then\;h_{3}=h_{1}∛3$$

Continuing step by step to consider the sequential movement of truncated cone layers from the gas source, we will thus obtain that in time *t = n* the gas will propagate to a distance

(5) $$r_{n}=r_{1}∛n\;or\;h_{n}=h_{1}∛n$$

There will come a moment when the next layer (the base of the truncated cone) reaches the location of the gas sensors at the level of the room ceiling. Further, the gas area will begin to spread along the ceiling in concentric circles. The cloud boundary will reach one or more gas sensors and the security system will signal the presence of gas. Let the angle of the cone apex be *α*°, (expressed in angles or radians). The volume of the cone *V* through the height of the cone *h* and the angle *α*, at the first unit time.

*V*_1_ = 1/3 *π h*_1_^3^
*tg*^2^
*α*,

Comparing the expressions for volumes at the first and second unit’s time, we obtain that the second height is expressed in terms of the first height as

$$h_{2}=h_{1}∛2,\;but\;in\;general\;h_{n}=h_{1}∛n$$

Thus, the increase in height, and hence the rate of gas rise, does not depend on the angle at the cone base at a constant power of the gas source. The expected vertical gas velocity is equal to the difference in the heights of the gas cloud cones per unit time:

(6) $${v_{n}=h}_{n+1}-h_{n}$$

Thus, the room heights and the intensity of the gas source (the number of molecules that flow out per unit of time and occupy a certain volume) are given. It is necessary to calculate the time for the gas cloud to reach the room ceiling at the level of the gas sensors, and the time it takes for it to reach one of the sensors.

Since each height increment is created per unit time, the height increment is numerically equal to the upward gas velocity, which is shown in Formula (6).

The time to reach the room ceiling is equal to the number of unit layers of the cone *t = n*, at which the total cone height of the gas cloud *h_n_* becomes equal to the height of the room *H*. The upward velocity of the gas, *v*, will decrease in proportion to the increase in the area of the cone layer’s base.

Height *H*, the level of gas sensors location will be reached in time *t*, equal to the number of layers of gas *n* when it propagates to the ceiling *t = n*

(7) $$H_{n}=r_{1}\sqrt[{3}]{t}=r_{1}\sqrt[{3}]{n}$$

The volume of the initial gas emission depends on the magnitude of the angle of the base of the cone. If we assume that the angle at the base of the cone is 60 degrees, then the initial volume will be three times less than for an angle of 90 degrees. The radius of the gas cloud on the ceiling will be smaller. The speed of movement along the ceiling to the locations of gas sensors will also change.

Consider an example of calculating the time and speed of gas propagation in a room. Height values are selected taking into account the technological features of the protected premises.

Let the room height be 4 m. Let us take a second as a unit time. The gas ejection cone in the first second has a height of 1 m, the cone apex angle is equal to the right angle, 90°, i.e., the cone radius is equal to the height, *r = h*, the gas source has a constant intensity. Table 1 gives the characteristics of the cloud: the cone height over time, the velocity of the upward gas propagation, and the volume dynamics of the gas cloud. It shows the time during which, under given conditions, the gas cloud reaches the height of the ceiling, and the accumulated volume of gas in the room.

At 64 s, the gas cloud reaches the ceiling. The accumulated volume of gas in the room is the circle radius and the cone base is equal to the cone height, therefore, it is 4 m, and then the gas begins to spread horizontally under the ceiling. A similar calculation shows that at a room height of 6 m, the gas reaches the ceiling at 220 s from the start of the ejection.

#### 2.3.2. Gas Movement from the Place of Reaching the Ceiling to the Sensors

After raising the gas cloud to the ceiling, we obtain a circle at the top, the dimensions of which may be less than the distance between the sensors. The gas will spread across the ceiling until it reaches the sensor, which will generate an alarm, i.e., report the danger.

Suppose that when moving along the ceiling, the thickness of the gas layer is constant, and is equal to the height of the last layer of the truncated cone that reached the ceiling. In Table 1, the thickness of the gas layer at the ceiling was equal to 0.0207 m. For each subsequent period of time (second), a ring of gas diverges along the ceiling, the volume of which is equal to the previous volumes (assuming a constant intensity of the gas source). When the ceiling is reached, the area of the circle *S_n_* is equal (if the angle of the cone is 90°):

*S_n_* = *π r_n_*^2^ = *π h_n_*^2^ = *π H*^2^ (8)

This is a two-dimensional case of the pattern that is considered in the analysis of the rise of a cloud of gas to the ceiling. That is, each ring that spreads across the ceiling has the same area as the circle, the base of the cone, and a cloud of gas that has risen to the ceiling.

In the second period, the gas will spread further, occupying the next ring. The area of the second ring, as well as subsequent rings, is equal to the area of the base of the cone. From here we find the rate of gas propagation. For the second period of time, gas will occupy an area of *S_n+_*_1_.

*S_n_*_+1_ = *π r_n_*_+1_^2^

Since the power of the source is constant, the increment of the area of the circle, the ring, *dS_n+_*_1_, occupied by the gas for the second unit of time, is equal to the area *S*_1_, occupied by the gas for the first unit of time. The area of the ring is equal to the difference between the area *S_n+_*_1_ = *π* *r_n+_*_1_^2^ and the area *S_n_* = *π* *r_n_*^2^. Then, we obtain

*d S_n_*_+1_ = *S_n_*_+1_ − *S_n_* = *π r_n_*_+1_^2^ − *π r_n_*^2^ = *S_n_*, (9)

where *dS_n+_*_1_ is the increment of the gas propagation area for the second period of time of movement along the ceiling, from which, we obtain *S_n+_*_1_ *=* 2 *S_n_ =* 2 *π* *r_n_*^2^. It follows that the radius of the ring *r_n+_*_1_ is

(10) $$r_{n+1}=r_{n}\sqrt{2}$$

For similar reasoning, we obtain, in the third period of time, the gas that will occupy a ring of radius *r_n+_*_2_.

(11) $$r_{n+2}=r_{n}\sqrt{3}$$

Continuing to consider the sequential propagation of the rings, we obtain, during the time *t = n +* m, the gas that will propagate over a distance

(12) $$r_{n+m}=r_{n}\sqrt{m}$$

Two points in time are of interest. The first is when the radius of the ring exceeds the distance between the sensors. From this point on, gas detection is guaranteed. The second point is when the radius of the ring reaches the size of the room. From this point on, we can assume that the room is filled with gas.

Table 2 shows the calculation of the gas propagation on the ceiling before reaching the sensor. Further, the movement of gas to the boundaries of a square room, with a side of 15 m.

The first column shows the time elapsed since the beginning of the movement of the gas cloud on the ceiling. The radius of the ring is calculated by Formula (12). The distance between the sensors is *A =* 6 m. In this case, when the radius of the ring is equal to *A* at 40 s, the volume of gas will be 109 m^3^. The gas reaches a distance of 6 m to the walls of the room in 80 s. At the distance between the sensors *A =* 5, the radius of the ring is equal to *A* at 25 s, the volume of gas will then be 93 m^3^.

The intensity of the source determines the rate of propagation of the gas. At the same time, the higher the velocity of the gas, the faster it will reach the sensor layer and will be registered. In this respect, the power of the source and the increase in the dangerous volume of gas are not directly proportional. This dependence can be widely neglected. The amount of gas in the hemisphere under the ceiling is proportional to the volume and density of the gas. The assumption that the gas propagates in a hemisphere of the same density is sufficient to ensure a level of safety, since it is a stronger condition than would be necessary if the density of the gas decreases as it moves away from the source.

#### 2.3.3. Selecting Sensor Locations

**Figure 2:**
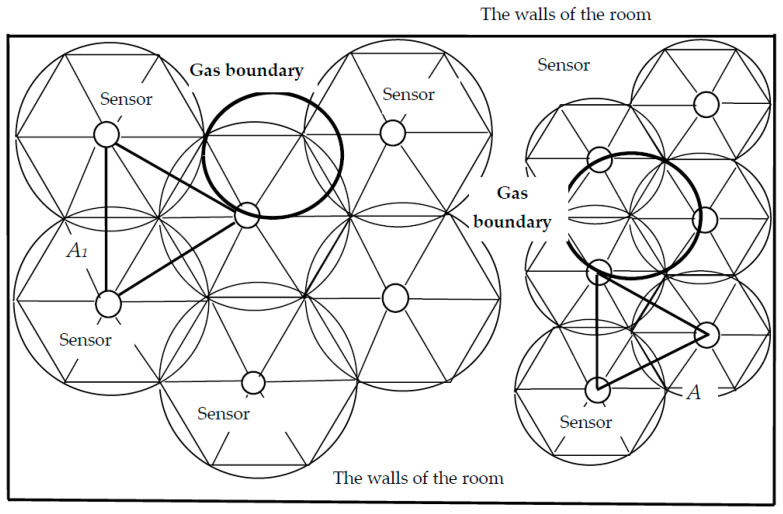
Views from above. Variants of hexagonal arrangement of sensors with a distance between them *A*_1_ and *A*_2_. The black circle represents the boundary of the area of gas that has risen to the ceiling.

It is necessary to choose the location of sensors in the protected area, which will ensure safety and will be economically feasible. The hexagonal arrangement of the sensors provides the densest coverage of the protected area without gaps; the distances between the sensors form equilateral triangles, with the side that we denote through *A*. Figure 2 shows two variants of the hexagonal arrangement of sensors: on the left with a lower frequency, with a distance between sensors *A*_1_; on the right with a higher frequency, with a distance *A*_2_. The black circle corresponds to the boundary of the gas area, which has risen to the top and spreads along the ceiling.

With a more frequent arrangement of sensors, gas is detected earlier, with a smaller volume of gas emissions. The ceiling area is covered with sensors without gaps, except for areas near the walls of the room. For complete closure, additional sensors can be placed near the walls. At the same time, when the gas rises along the walls, the pressure will more actively displace its molecules into the inner regions of the room, into the zones of action of the sensors. Thus, at the walls and in the corners of the room, the gas reaches the sensors faster than in the centre of the room.

**Figure 3:**
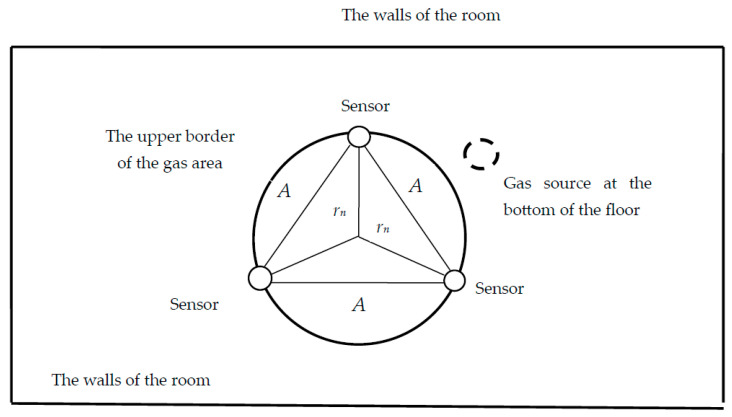
Gas propagation area touches sensors on the ceiling, top view.

Figure 3 shows a circle of gas on the ceiling of the room, which is described around a triangle of sensors arranged in a hexagonal pattern. This is an element of the situation shown in Figure 2. The figure shows the most unfavourable situation that occurs when the gas region reaches the ceiling in the centre of the triangle, at the vertices of which the sensors are located.

The position of the gas source under the sensors corresponds to the largest volume of gas accumulated in the room. Therefore, if the gas reaches the level of the sensors at any other point, then the gas leak notification will be received with smaller volumes of gas in the room. The sensors form an equilateral triangle, the side of which is equal to *A*—the distance between the sensors. The radius of the described circle L, which determines the area of gas propagation along the ceiling, is

(13) $$r_{n}=A\frac{\surd 3}{3}$$

The mass *m_g_* of a given volume of gas, as shown in (1), with guaranteed registration at a constant density *q* (which can be considered the maximum, i.e., the upper estimate of the mass of the gas, taking into account diffusion and pressure change)

*m_g max_ = V_g_ q* (14)

In this case, the minimum volume of gas *V_min_* = *V_up_* for registration occurs when the gas cloud reaches the ceiling height *H*, (the level of the sensors). Its mass is equal to

*m_g min_ = V_min_ q =* 1/3 *π r_n_*^2^ *H q* (15)

When the gas reaches one of the sensors, a message will appear indicating the presence of gas. The message will appear as a signal on the operator’s monitor, which shows the location of all sensors. The localization of the signals will indicate the location of the danger. At the same time, the signals are sent to the automated security system. In this system, the coordinates of the sensors that gave the signals are used to calculate the coordinates of the location of a possible gas source, and measures are taken to prevent the threat or eliminate the fire.

As the distance between sensors decreases, the number of sensors increases, and therefore, the cost of the safety system increases. At the same time, the volume of flammable gas that will have time to spread before it is registered is reduced.

Let us consider the *X* and *Y* coordinates of the placement of sensors under the ceiling, and the dependence of the volume of gas on the density of filling by the sensors. Let the room have dimensions *T_x_* on the *X* axis and *T_y_* on the *Y* axis; i.e., the area of the room *S* is equal to

$$S=T_{x}\times T_{y}.$$

Let us calculate the coordinates of the sensors at a hexagonal location. The distance between the sensors is *A*. The sensor is located in the centre of a regular hexagon with side *C*. Let us start placing the sensors from the origin located in the lower left corner of the room.

The coordinates of the first sensor *D*_11_, located in the first row, are equal to

(16) $${D_{11}:\;x}_{11}=C=A\frac{\surd 3}{3};y_{11}=\frac{A}{2}\;;$$

The coordinates of the second sensor in the first row *D*_12_ are

(17) $${D_{12}:\;x}_{12}=x_{11}+3C=C+3C=4A\frac{\surd 3}{3};y_{12}=\frac{A}{2}$$

The coordinates of the *n*-th sensor *D*_1*n*_ in the first row are

(18) $${D_{1n}:\;x}_{1n}=x_{1,n-1}+3C=x_{1,n-1}+A\sqrt{3};y_{1,n-1}=\frac{A}{2}$$

The first row is filled, and it is necessary to consider the possibility and necessity of filling the remainder to the wall of the room, which is provided by

(19) $$x_{1,n-1}\leq T_{x}.$$

The coordinates of the first sensor *D*_12_, located in the second row, are

(20) $${D_{12}:\;x}_{21}=C+\frac{3}{2}C=\frac{5}{2}A\frac{\surd 3}{3};y_{21}=A$$

The coordinates of the *n*_2_-th sensor *D*_2*n*_, located in the second row, are

(21) $${D_{2n}:\;x}_{2,n}=x_{2,n-1}+3C=x_{2,n-1}+A\sqrt{3};y_{2,n}=A$$

The second row is filled, and it is necessary to consider the need to fill the space up to the wall of the room, which is provided by

(22) $$y_{2,n-1}+\frac{A}{2}\leq T_{y}.$$

Having filled the rows of sensors to the right and up, we solve the issues of closing the areas adjacent to the walls with sensors. Practice shows that no special control is required at the borders. The corners and walls direct the gas to the central part of the protected room, where the sensors are located.

The sum of the intervals between the sensors in each row, taking into account the initial distance, should not exceed the length of the room.

(23) $$dx=1+\frac{Tx}{3C}=1+\frac{Tx}{A\sqrt{3}}.$$

The number of such rows is *d_y_ =* 2*T_y_*/*A*, where *A*/2 is the distance between the rows. Thus, it is possible to obtain an estimate of the total number of sensors *d*

(24) $$d=dx\times dy=(1+\frac{Tx}{A\sqrt{3}})\frac{2Ty}{A}.$$

The considered example demonstrates the application of the developed method of gas sensor location to ensure the fire safety of industrial premises.

## 3. Results and Discussion

According to the developed method, practical calculations of examples of choosing the location of sensors and determining their number were carried out. The calculations were carried out using a program for working with Microsoft Excel spreadsheets. Considering the choice of the location of the sensors depending on the permissible volume of gas in the protected room, the maximum allowable volume of gas in the room is denoted by *V_m_*.

As shown in Table 1, the volume of the gas cloud when the height of 4 m is reached will be *V_m_* = 67.02 m^3^. The radius of the base of the cone, with the assumptions made, will be 4 m. This volume can be taken as the maximum permissible value, since at lower limit values, the gas will not yet reach the sensors.

Let the dimensions of the room be 20 m by 10 m: *T_x_* = 20 m, *T_y_* = 10 m; the height of the room is *H* = 3 m.

**Figure 4:**
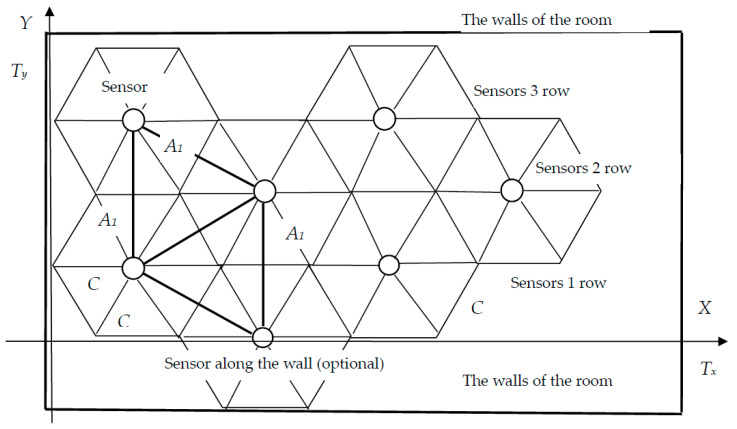
Hexagonal arrangement of sensors with the distance between them *A*_1_.

Under these conditions, according to Formulas (16)–(24) and Figure 4, the location of the sensors is determined. This arrangement will provide full coverage of the surface of the room, and provides control of potential gas emissions. If *r_n_* = 3 m, then, according to Formula (13), *A* = 5.2 m. Let the sensors be located no further than 4.34 m from each other. Let us calculate the coordinates of the sensors and their number.

The coordinates of the first sensor *D*_11_, located in the first row, are equal to

(25) $${D_{11}:\;x}_{11}=C=2.5;y_{11}=\frac{A}{2}=2.17\;$$

The coordinates of the second sensor in the first row *D*_12_ are

(26) $${D_{12}:\;x}_{12}=x_{11}+3C=4\;C=10;y_{12}=\frac{A}{2}=2.17\;$$

The coordinates of the third sensor *D*_13_ in the first row are

(27) $${D_{13}:\;x}_{13}=x_{2}+3C=7C=17.5;y_{13}=\frac{A}{2}=2.17$$

Since *T_x_* − *x*_13_ = 20 − 17.5 = 2.5, which is less than 3*C* = 7.5, i.e., the step of placement of sensors, the first row is filled. You can consider the need to fill the remainder to the wall of the room. There are three sensors here.

The coordinates of the first sensor *D*_21_, located in the second row, are equal to

(28) $${D_{21}:\;x}_{21}=C+\frac{3}{2}C=\frac{5}{2}C=6.25;y_{21}=A=4.34\;$$

The coordinates of the 2nd sensor *D*_22_, located in the second row, are

(29) $$D_{22}:\;x_{22}=x_{21}+3C=13.75,\;y_{22}=A=4.34$$

Since *T_x_* − *x*_22_ = 20 − 13.75 = 6.25, which is less than 3*C* = 7.5, i.e., the step of placement of sensors, the second row is also filled, and it is necessary to consider the need to fill the remainder to the wall of the room. There are two sensors here.

The coordinates of the sensors in the third row differ only vertically, i.e., along the *y*-axis. The coordinates of the first sensor *D*_31_, located in the third row, are equal to

(30) $$D_{31}:\;x_{31}=C=2.5;y_{31}=\frac{3}{2}\;A=2.17+4.34=6.51;$$

The coordinates of the second sensor in the third row *D*_32_ are equal to

(31) $${D_{32}:\;x}_{32}=x_{31}+3L=4\;C=10;y_{32}=\frac{3}{2}\;A=6.51\;$$

The coordinates of the third sensor *D*_33_ in the third row are

(32) $${D_{33}:\;x}_{33}=x_{2}+3C=7\;C=17.5;y_{33}=\frac{3}{2}\;A=6.51\;$$

Since *T_x_* − *x*_33_ = 20 − 17.5 = 2.5, which is less than 3*C* = 7.5, i.e., the step of placement of sensors, the third row is filled. You can consider the need to fill the remainder to the wall of the room. There are three sensors here.

The coordinates of the sensors in the fourth row differ only along the y-axis. The coordinates of the first sensor *D*_41_, located in the fourth row, are equal to

(33) $$D_{41}:\;x_{41}=C+\frac{3}{2}C=\frac{5}{2}C=6.25;y_{41}=2A=8.68$$

The coordinates of the second sensor *D*_42_, located in the fourth row, are equal to

(34) $${D_{42}:\;x}_{42}=x_{41}+3C=13.75,\;y_{42}=2A=8.68$$

Since *T_x_* − *x*_42_ = 20 − 13.75 = 6.25, which is less than 3*C* = 7.5, i.e., the step of placement of sensors, the fourth row is also filled, and it is necessary to consider the need to fill the remainder to the wall of the room. There are two sensors here.

Since *T_y_* − *y*_42_ = 10 − 8.68 = 1.32, which is less than *A*/2 = 2.17, i.e., the step of placement of sensors along the y-axis, the ceiling surface is filled and the problem is solved.

In principle, it is possible to consider the need to apply sensors to the rest of the ceiling and to the wall of the room.

(35) $$y_{2,n-1}+\frac{A}{2}\leq T_{y}.$$

Having filled the rows of sensors to the right and up, until the boundaries of the room are reached, we solve the issues of closing the areas adjacent to the walls with sensors.

The number of sensors in a room is determined by the size of the room and the distance between the sensors. The number of sensors in two rows of three and in two rows of two equal a total of 10 detector sensors needed.

**Figure 5:**
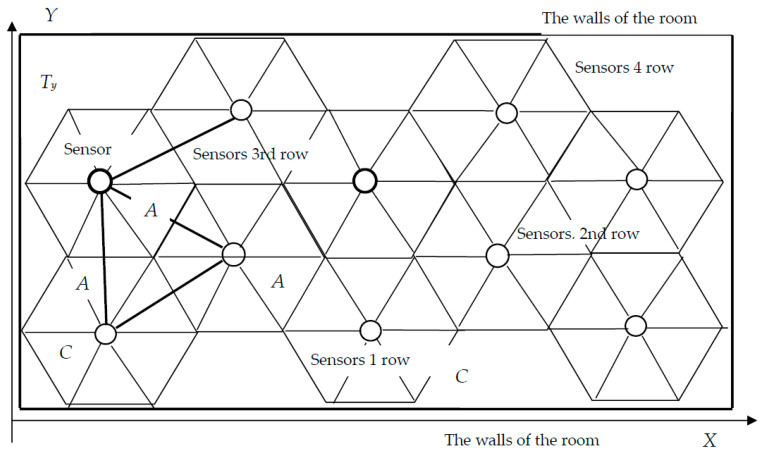
Location of sensors on the ceiling of a room 20 m long, 10 m wide, with a height of 3 m. Overhead view.

The arrangement of sensors in four rows on the ceiling of the room is schematically shown in Figure 5. This can be considered as a framework that, if necessary, can be completely moved to give the system greater symmetry.

The number of sensors in the room is determined by the size of the room, the permissible volume of hazardous gas, and the sensitivity of the sensors. These parameters determine the distance between the sensors to the greatest extent.

The considered example represents the rational placement of sensors that register the appearance of a dangerous gas. The developed method makes it possible to adjust the placement of sensors depending on the maximum permissible volume of gas and the sensitivity of the sensors used. The method makes it possible to control the layout of sensors depending on the volume of protected premises, types of production, the creation of new sensors, changes in the composition of hazardous gas in different technological processes.

Next, it is necessary to calculate the localization (coordinates of a possible place) of the gas emission according to the coordinates of the sensors that signal the registration of a hazardous substance in the room. This method provides a calculation of the coordinates of the gas source from the coordinates of the triggered sensors. Knowing the coordinates of all sensors, the coordinates of the sensors that gave the alarm, and the formula for determining the volume of the gas cloud under these sensors, it is possible to assess the localization of the source of hazardous gas in the protected premises. This increases the efficiency of the fire safety system.

The method provides the calculation of the rational placement of sensors when changing conditions: the height and size of the room, increasing the sensitivity of the sensors, the requirements for the maximum permissible volumes of recorded gases, etc.

The considered method assumes that ideal conditions are created in the room for monitoring the air without the influence of wind caused by the fan (air conditioner). The force of the wind (ventilation) creates the movement of air and gas in the horizontal direction. The lifting force acts on the gas (of the type in question) vertically. The forces act independently, so the gas molecules will rise to the ceiling, thus, to the sensors. The lifting time will not change significantly, but the place where the ceiling is reached will change.

The problem can be solved by entering into the calculation a correction factor that shifts the “gas cone” and the layout of the sensors in the direction of the wind. At a certain high-air-flow rate, gas molecules can be outside the room before they reach the ceiling. Then, you need to add sensors at the outlet of the air flow from the room.

Production rooms of a large size and complex configuration can be divided into small rooms. For each part, apply the developed method of sensor placement. The problem is to connect the boundaries of the individual parts. To do this, you can apply the method of network models, for which the author has developed algorithms for calculating in parts, presented in [24,25]. This will require, of course, additional work, such as the creation of a network model of this subject area. In the future, it is planned to conduct such a study.

## 4. Conclusions

One of the important problems of maintaining the operation of industrial enterprises is to ensure fire safety. It is important to detect a potential fire before ignition, at the stage of when deviation beings from the normal technological process. Indicators of an emergency situation are various gases, an increase in the concentration of which indicates the beginning of a dangerous process. The creation and use of gas sensors makes it possible to detect small changes in the concentrations of hazardous gases. The article presents a mathematical method for choosing the location and number of sensors that solve the problem of gas control. The natural physical approach will change from the standpoint of analyzing the gas flow from the source to the locations of sensors in the premises of enterprises.

The developed mathematical model of the rational location of gas detectors on H_2_ and CO for early detection of fire (accident) is presented. The model-based method for calculating the location of gas analyzers makes it possible to diagnose and predict fire hazard at technological facilities based on gas control, and compare the obtained values with the maximum permissible values. The calculation of the conditions for the rational placement of gas detectors for different heights of technological premises is carried out.

The method made it possible to determine the rational placement of sensors that ensure timely registration of gas, taking into account economic feasibility. The resulting solution can be algorithmically converted for use in other conditions, when changing the size, height, shape of the room, and when changing the norms of permissible volumes of hazardous gas. The method provides a change in decisions with the appearance of more sensitive sensors. The method is also applicable for technologies where other types of gases are indicators. It is planned to develop a mathematical program for an automated fire protection control system with an early fire detection function based on H_2_ and CO gas control in technological design.

## Figures and Tables

**Table 1:** The geometrical characteristics of the gas cloud when rising to the ceiling.

Time Since the Beginning of the Ejection, s	The Height of the Gas Cone at Each Moment	Gas Cloud Rise Rate	Room Height	The Difference between the Heights of the Room and the Cone	Accumulated Volume of Gas Cone
*t*, s	*r = h*, m	*v*, m/sec	*H*, m	*H* − *ht*	*V*, m^3^
1	1.00	1.0000	4.0	3.00	1.05
10	2.15	0.4445	4.0	1.85	10.47
20	2.71	0.2482	4.0	1.29	20.94
50	3.68	0.1271	4.0	0.32	52.36
60	3.91	0.1119	4.0	0.09	62.83
64	4.00	0.0209	4.0	0.00	67.02
65	4.02	0.0207	4.0	−0.02	68.03

**Table 2:** The geometrical characteristics when the gas cloud moves along the ceiling to the sensors.

Time of Movement on the Ceiling, s	The Radius of the Ring *r_i_,* m	The Speed of the Gas Ring	Distance between Sensors, *A*, m	*A* − *r_i_*	Gas Cloud Volume *V + Vup*	The Length of the Walls of the Room, *L*, m	Distance from the Gas Ring to the Walls *L* − *r_i_*
5	2.24	2.2361	6.0	3.76	72.26	15.00	12.76
10	3.16	0.9262	6.0	2.84	77.49	15.00	11.84
20	4.47	0.5992	6.0	1.53	87.96	15.00	10.53
30	5.48	0.4772	6.0	0.52	98.44	15.00	9.52
40	6.32	0.4085	6.0	−0.32	108.91	15.00	8.68
50	7.07	0.3629	6.0	−1.07	119.38	15.00	7.93
60	7.75	0.3298	6.0	−1.75	129.85	15.00	7.25
70	8.37	0.3043	6.0	−2.37	136.14	15.00	6.63
80	8.94	0.2840	6.0	−2.94	138.23	15.00	6.06

## Data Availability

Not applicable.
